# Evaluating the validity of animal models of mental disorder: from modeling syndromes to modeling endophenotypes

**DOI:** 10.1007/s40656-022-00537-4

**Published:** 2022-11-10

**Authors:** Hein van den Berg

**Affiliations:** Department of Philosophy, Institute for Logic, Language and Computation, University of Amsterdam, Postbus 94201 1090 GE, Amsterdam, The Netherlands

**Keywords:** Animal models, Mental disorders, Validity, Endophenotype

## Abstract

This paper provides a historical analysis of a shift in the way animal models of mental disorders were conceptualized: the shift from the mid-twentieth-century view, adopted by some, that animal models model syndromes classified in manuals such as the *Diagnostic and Statistical Manual of Mental Disorders* (*DSM*), to the later widespread view that animal models model component parts of psychiatric syndromes. I argue that in the middle of the twentieth century the attempt to maximize the face validity of animal models sometimes led to the pursuit of the ideal of an animal model that represented a behaviorally defined psychiatric syndrome as described in manuals such as the *DSM*. I show how developments within psychiatric genetics and related criticism of the *DSM* in the 1990s and 2000s led to the rejection of this ideal and how researchers in the first decade of the twenty-first century came to believe that animal models of mental disorders should model component parts of mental disorders, adopting a so-called endophenotype approach.

## Introduction

Animal models of mental disorder represent aspects of human psychopathology. Researchers on animal models have questioned how *valid* animal models of mental disorders can be constructed. Ankeny et al., (2014), investigating animal models of alcohol addiction, note that “one of the main questions of validity in this field concerns whether animals can be used to understand the ‘uniquely human’ phenomenon of alcohol addiction” (p. 493). This question is pressing since there is evidence that alcoholism is peculiar to humans (ibid.). In this paper, I focus on the disorder of depression and investigate how researchers on animal models of mental disorders have reflected on the validity of their models in the middle of the twentieth-century and the first decade of the twenty-first century.

Ankeny & Leonelli (2011) distinguish between the “representational scope” and “representational target” of experimental organisms (p. 315). The representational scope describes “how extensively the results of research with a particular experimental organism (a specimen or token) can be projected onto a wider group of organisms (a type)” (ibid.). By contrast, the representational target indicates “the phenomena to be explored through the use of the experimental organism” (ibid.). Both the representational scope and the representational target can vary during research (ibid.). This paper provides a historical study of animal models of mental disorders that illustrates the shifting representational target of such models. I analyze a shift in the way animal models of mental disorders were conceptualized: the shift from the view, adopted by some in the middle of the twentieth century, that animal models represent syndromes classified in manuals such as the *Diagnostic and Statistical Manual of Mental Disorders* (*DSM*), to the widespread early twenty-first century view that animal models model component parts of psychiatric syndromes, a view encapsulated in the endophenotype approach. I argue that in the middle of the twentieth century the attempt to maximize the face validity of animal models sometimes led to the pursuit of the ideal of an animal model representing a behaviorally defined psychiatric syndrome as described in manuals such as the *DSM.* I show that developments within psychiatric genetics and related criticisms of the *DSM* in the 1990s and the 2000s led to the rejection of this ideal.

My paper builds on the work of Nelson (2018), which provides an ethnographic study of twenty-first century research on animal models (mice) of mental disorders. Nelson writes that animal researchers almost always break down behaviors into smaller units for analysis (2018, p. 23. See also Ankeny et al., 2014, p. 496). This method is described by Nelson as a pragmatic methodological and experimental program. She contrasts this methodological stance to the endophenotype project, which she briefly describes as an ontological project concerned with providing a new biological basis for psychiatric classification (2018, p. 29). In contrast to Nelson, I adopt a history of ideas approach that discusses both twentieth-century, which Nelson does not discuss, and twenty-first century views on the validity of animal models of mental disorder. I show how developments within psychiatric genetics and the increasing criticism of the *DSM* in the 1990s and 2000s led to a rejection of the ideal that animal models must represent *DSM* classifications. On this basis, I explain the popularity of the twenty-first century endophenotype approach, which Nelson does not extensively discuss.^1^ The main novelty of the paper consists in adopting a broad historical perspective on the history of animal modeling and describing the shift from the mid-twentieth century view that models should capture as many aspects of a behavior or disorder as possible to the contemporary view that we should model component parts of disorders. There is little to no work that ties these periods together and explains this transition. The present paper is supposed to fill this gap.

Throughout this paper, I will mainly focus on animal models of *depression*. This is because the conceptual shift that I wish to describe, i.e., the shift from conceptualizing animal models as representing entire syndromes to adopting the endophenotype approach, can be profitably understood on the basis of a focused analysis of animal models of depression. Criteria for validity of animal models of mental disorders were initially formulated for models of depression and some of the pioneers of work within the field of animal models of mental disorders were engaged with animal models of depression. However, it is important to note that the conceptual shift that I describe in this paper is not limited to animal models of depression. The search for endophenotypes occurs within research on multiple mental disorders, such as bipolar disorder and schizophrenia, to name just a few. Hence, many features of my analysis will generalize to many different animal models of mental disorder.

In the second section, I describe the criteria on the basis of which animal models of mental disorder are validated. In the third section, I illustrate how the cooperation between psychoanalysis, comparative psychology, and ethology led to the creation of animal models of depression (3.1). I further show how research on psychopharmacology impacted the creation of animal models of depression in the 1950s and 1960s (3.2). I argue in these sections and in Sect. (3.3) that mid-twentieth-century research on animal models of mental disorders was sometimes characterized by the view that animal models model psychiatric syndromes listed in manuals such as the *DSM*. The fourth section describes the developments in psychiatric genetics in the 1990s and 2000s and the related criticism of the *DSM* in psychiatry in this period, developments that paved the way for the endophenotype approach to animal modeling . In Section 5, I describe the endophenotype approach and the arguments researchers give for adopting this approach.

## Validating animal models of mental disorders: predictive validity, face validity, and construct validity

McKinney, a pioneer in the field of animal models of depression and a writer of one of the few textbooks on comparative psychiatry, defined animal models as “experimental preparations developed in one species for the purpose of studying phenomena occurring in another species” (McKinney, 1988, p. 20). Today, it is customary to validate animal models of mental disorders according to Willner’s 1984 criteria of predictive validity, face validity, and construct validity (on the history of these criteria, Abramson & Seligman, 1977, Belzung & Lemoine, 2011, Van der Staay et al., 2009). In the following I explain these criteria.

According to Willner, predictive validity is assessed “by whether a model correctly identifies (1) antidepressant treatments of pharmacologically diverse types, (2) without making errors of omission (3) or commission, (4) and whether potency in the model correlates with clinical potency” (Willner, 1984, p. 1). Condition (2) means that a model should not show false-negative results, i.e., a model should not fail to identify drugs known to be effective (Willner, 1986, p. 680). Condition (3) states that a model should not show false-positive results, i.e., a model should not incorrectly identify drugs known to be ineffective (ibid.).

Willner illustrates the criterion of predictive validity with the model of learned helplessness (Willner, 1986). In the experiment of Seligman, dogs were exposed in a first trial to electric shocks which they could not escape, while other dogs were exposed to shocks that they could escape (Willner, 1986, p. 679). In later trials, where all dogs could escape, the dogs that had been subjected to inescapable shocks passively accepted the shocks (ibid.). The interpretation of this experiment was that these dogs had learned that anything they did would be ineffective (ibid.). According to the learned helplessness theory of depression, “the cause of depression is the belief that a person is helpless” (ibid.). We can view the animal model of learned helplessness as an animal model of depression.

The animal model of learned helplessness can be evaluated according to the criterion of predictive validity. As Willner notes (1986, p. 681), treatment with various antidepressants has been shown to be effective in reversing learned helplessness. Hence, the animal model of learned helplessness correctly identifies (1) antidepressant treatments of pharmacologically diverse types, and does not (as far as we know) (2) fail to identify drugs known to be effective against depression. In addition, treatment with drugs known to be ineffective against depression was not effective in reversing helplessness (Willner, 1984, p.6). Hence, the model did not show (3) false positive results, even if not all potential false-positives were tested. Finally, Willner notes that the correlation test (4) cannot be applied since the drugs studied did not differ greatly in their potency (ibid.).

Willner’s criterion of *face validity* is construed as follows: “face validity is assessed by whether antidepressant effects are only present on, or are potentiated by, chronic administration (1), and whether the model resembles depression in a number of respects (2), which are specific to depression (3), and do actually coexist in a specific sub-group of depressions (4); also, the model should not show features which are not seen clinically” (Willner, 1984, p. 1). In 1986, Willner noted that similarity of symptoms between the model and the human condition is the most basic criterion of face validity (Willner, 1986, p. 682).

We can again illustrate Willner’s criterion of face validity by reference to the animal model of learned helplessness. Willner notes that helpless animals exhibit many symptoms which are analogous to symptoms of depressed humans (Willner, 1984, p. 6). Hence, the model scores relatively well on criterion (2) of face validity. However, Willner notes that it is not clear if there is a specific subgroup of depressions to which the symptoms of learned helplessness are similar (Willner, 1986, p. 682). Finally, Willner notes that learned helplessness does not score well on the specificity criterion (3), according to which the model exhibits symptoms that are specific to depression. The reason is that learned helplessness is also associated with other psychiatric disorders (Willner, 1984, p.6).

Willner argues that construct validity is assessed by “whether both the behavior in the model (1) and the features of depression being modeled (2) can be unambiguously interpreted, and are homologous (3), and whether the feature being modeled stands in an established empirical (4) and theoretical (5) relationship to depression” (Willner, 1984, p.1). As Belzung & Lemoine (2011) note, in a later paper Willner simply describes construct validity as an attempt to establish similarity both at the level of the behavioral and/or cognitive dysfunctional process, and to establish similarity at the level of etiology (Willner & Mitchell, 2002).

The criteria of construct validity are illustrated by means of separation models. Infant monkeys separated from their mother exhibit symptoms that are analogous to the symptoms exhibited in the state of anaclitic depression, which is ascribed to children separated from their mothers (Willner, 1984, pp. 8–9). The primates and human infants thus exhibit similar symptoms which are presumably due to the same etiology. Hence, there is a homology (3) between primate depression and anaclitic depression. The behavior of the model and the features of depression being modeled can also be unambiguously interpreted (1, 2). However, Willner also notes that “no consensus exists as to the relationship between infantile anaclitic depression and depression in adults” and that even “the assumption that separation from a loved one is a significant cause of adult depression” has been questioned (Willner, 1984, p.9).^2^

In the rest of this paper, I refer to Willner’s criteria of predictive, face, and construct validity when discussing the validity of animal models. In the next section, I provide historical case studies that demonstrate that in the mid-twentieth century animal models of mental disorders were sometimes taken to model psychiatric syndromes as classified in the *DSM*.

## Animal models of mental disorders in the middle of the twentieth century: maximizing face validity

Ramsden (2018) has described that in the early twentieth century American psychiatrists and psychologists turned to the works of Ivan Pavlov in order to build an objective approach to psychiatry. Ramsden notes that William T. McKinney, himself a pioneer in the field of animal models of mental disorder, identified Pavlov as the forefather of his own approach (Ramsden, 2018, p.147). Pavlov’s ideas and methods were taken up in the United States in the 1920s and 1930s through researchers such as W. Horsley Grantt, Howard Liddell and Jules Masserman, who studied experimental neurosis (Ibid., p. 148. See for an overview of comparative psychiatry from 1923 to 1962, Kirk & Ramsden, 2018). In the 1960s, McKinney himself wrote foundational works on animal models of depression. In their pioneering paper of 1969, entitled “Animal Model of Depression”, McKinney and Bunney argued for “the need for an experimental model of ‘depression’ and wished to “review pertinent evidence from a variety of fields which points to the feasibility of such a model” (1969, p. 240).

We can understand the state of psychiatric knowledge during the time McKinney and Bunney wrote their article by considering the *Diagnostic and Statistical Manual of Mental Disorders* at the time. These were the *DSM-I* of 1952 and the *DSM-II* of 1968. As Grob (1991, p. 427) has stressed, the *DSM-I* was the result of post-wartime psychiatry which was dominated by psychodynamic and psychoanalytic concepts. The`*DSM-II* maintained the psychodynamic and psychoanalytic perspective of the first *DSM*. Hence, McKinney and Bunney wrote on animal models of depression against the background of a psychiatric profession dominated by the psychoanalytic and psychodynamic perspectives. In what follows I argue that it was the cooperation between psychoanalysis, ethology, and comparative psychology that led to the creation of animal models of depression.

As evidence for the feasibility of an animal model of depression, McKinney and Bunney referred to separation experiments with monkeys. They mentioned several studies of Harlow and associates, in which, for example, “they separated four mother-infant pairs of rhesus monkeys for a three week period” (McKinney & Bunney, 1969, p. 241). McKinney and Bunney also pointed to similar studies from the ethologist Robert Hinde and co-workers and to studies from Kaufman and Rosenblum (ibid.). All these studies induced depressive behavioral changes in the young monkeys and provided evidence for the possibility of creating an experimental animal model of depression. Below we will look at some of these separation experiments in their historical context in order to determine how they modeled depression.

### Separation experiments and animal models of depression

The separation experiments of Harlow and associates built on the work of John Bowlby (1907–1990). Bowlby was a clinician trained in psychoanalysis (Van der Horst et al., 2008, p. 371). He is famous for introducing the idea that the separation of children from their caregivers can lead to mental disturbances (ibid., pp. 371–372). In 1951, Bowlby became acquainted with the science of ethology. He read the works of Konrad Lorenz (1903–1989), Nikolaas Tinbergen (1907–1988), and Robert Hinde (1923–2016) (van der Horst, 2011, Chap. 4). In 1957, Bowlby published an article entitled “An Ethological Approach to Research on Child Development”. This article presented ethology as a discipline that could render psychoanalysis more scientific. While synthesizing psychoanalysis and ethology, Bowlby formed a close relationship with the comparative psychologist Harry Harlow, famous for his work on separation experiments in monkeys (Van der Horst et al., 2008). As Van der Horst et al. describe, Bowlby used Harlow’s work with rhesus monkeys as support for his attachment theory, whereas Harlow “tried to model his rhesus work to support Bowlby’s theoretical framework” (2008, p. 371. On Harlow, see also Guerrini, 2003).

Bowlby applied ethology to child development research (van der Horst, 2011, Chap. 4). In 1958, Bowlby put forward the idea that the attachment behavior which we observe in babies is made up of hereditary “component instinctual responses”, including sucking, clinging and following (Bowlby, 1958, p. 351). These instinctual responses serve the function of binding the child to the mother (ibid.). In a later paper, dealing with separation anxiety, Bowlby recorded observations of children who, when admitted to a hospital, were separated from their mothers. These children exhibited a sequence of behavior which consisted of *protest*, *despair* and *detachment* (Bowlby, 1960b, p. 90). During the protest phase the children were distressed and tried to recapture the mother (ibid.). During the despair phase, the behavior of the children suggested “increased hopelessness” (ibid.). Their movements diminished and they became withdrawn and inactive (ibid.). Finally, during the detachment phase, when the mothers visited, there was “a striking absence of the behaviour characteristic of the strong attachment normal at this age” (Bowlby, 1960b, p. 90). Bowlby conceptualized protest, despair, and detachment as behaviors resulting from the activation of instincts coupled with the absence of the mother (Bowlby, 1960a).

The psychiatric theory that Bowlby drew on in articulating his theory of separation anxiety was psychoanalytic. According to Freud’s theory, as Bowlby explains, anxiety is a result of the transformation into anxiety of “sexual excitation of somatic origin which cannot be discharged” (Bowlby, 1960b, p. 93). The anxiety of infants separated from a loved person is an example of this (Ibid.). The theory of primary anxiety held that the anxiety is a primary response that results simply from the rupture of the attachment to the mother (ibid.). Bowlby took his own theory of separation anxiety to be a version of this last theory.

Bowlby’s theory of separation anxiety was taken up by Seay, Hansen and Harlow, who studied mother-infant separation in monkeys (1962, see also Seay & Harlow, 1965). These authors reasoned that if, as Bowlby claimed, separation anxiety was the result of the activation of basic instinctual responses and the absence of a mother, a similar syndrome should develop in infant monkeys following separation from their mothers (Seay et al., 1962, p. 123). This hypothesis was tested by separating two pairs of infant rhesus monkeys from their four mothers (Seay et al., 1962, p. 125).

Seay et al. studied the infants during a pre-separation period, a separation period and a post-separation period when the infants were allowed to return to their mothers. Immediately after separation, the behavior of the infants included scampering, screeching and crying (Seay et al., 1962, pp. 126–127). These behaviors mirrored the behaviors of human infants during what Bowlby called the protest phase. After the protest phase, the behavior of the rhesus monkeys was characterized by decreased activity (Seay et al., 1962, p. 128). This behavior mirrored what Bowlby had called the despair phase of human infants. Post-separation behavior, when the monkey infants returned to their mothers, was characterized by the increase of infant-mother clinging and cradling (Seay et al., 1962, pp. 129–130). Seay et al. argued that their experiment provided results that were in accord with expectations based on the human separation syndrome described by Bowlby (ibid., p. 130). The separated monkeys went through what Bowlby had described as protest and despair phases, although there was no detachment phase.

Why did McKinney and Bunney take the separation experiments of Harlow and associates as evidence for the possibility of animal models of depression? McKinney & Bunney (1969, p. 240) distinguished between primary and secondary symptoms of the depressive syndrome. The primary symptoms consisted of “a despairing emotional state and a depressive mood” (ibid.). The secondary symptoms included such things as social withdrawal, psychomotor retardation, and weight loss. McKinney and Bunney understood the term “depression” in an operational sense referring to the secondary symptoms, i.e., to observable behavioral changes that are associated with depression in humans (ibid.). Insofar as mental disorders were understood in behavioral terms, McKinney and Bunney could point to behavioral similarities between psychiatric disorders and animal behavior to argue for the possibility of an animal model of depression. The methodology that McKinney and Bunny employed can be summarized as follows: we must have a clear behavioral description of a psychiatric syndrome, such as separation anxiety, and consequently we test whether animals exhibit analogous behavior. The animal model is thus taken to model a psychiatric syndrome and the validity of the animal model is partly tested by studying behavioral similarities between the animal model and the human depressive syndrome.

McKinney and Bunney also took the work of Kaufman & Rosenblum (1967) to support the idea of animal modeling. Whereas Harlow and associates utilized Bowlby’s theory of separation anxiety, Kaufman & Rosenblum (1967) compared the behavior of pigtail monkey infants separated from their mothers to the theory of anaclitic depression of the psychoanalyst René Spitz. René Arpád Spitz (1887–1974), a Hungarian-American psychoanalyst, is known for writing on the dangers of institutional child care and the separation of the child from their mother (van Rosmalen et al., 2012, p. 425).

In his ’Anaclitic Depression’ (1946), Spitz investigated what happened to infants in a nursery when separated from their mothers. Spitz noted that after separation the infants exhibited a syndrome composed of the following elements (i) “Apprehension, sadness, weepiness”; (ii) “lack of contact, rejection of environment, withdrawal”; (iii) “retardation of development, retardation of reaction to stimuli, slowness of movement, dejection, stupor”; (iv) “loss of appetite, refusal to eat, loss of weight”; (v) “insomnia” (Spitz, 1946, p. 316). The syndrome was called ‘anaclitic depression’.

Kaufman and Rosenblum related the behavior of pigtail monkey infants separated from their mothers to Spitz’ theory of anaclitic depression. One of the reasons for Kaufman & Rosenblum (1967) to adopt Spitz’ theory, as opposed to Bowlby’s theory of separation anxiety, was that infant monkeys did not seem to undergo Bowlby’s detachment phase when reunited with their mothers. The theory of anaclitic depression did not postulate a detachment phase but only described two stages that occurred when infants were separated from their mothers: apprehensive crying and depression (Kaufman & Rosenblum, 1967, p. 651). This account better fitted the data obtained by separation studies in monkeys.

After separating four pigtail infants from their mothers, Kaufman and Rosenblum observed loud screams by both mothers and infants and struggles to reunite (ibid., p. 654). After 24–36 h, the infant monkeys became depressed (Kaufman & Rosenblum, 1967, pp. 654–656). This depression lifted after five to six days and after the mother was returned. Kaufman and Rosenblum concluded that their observation of pigtail infants was in accord with what Spitz reported for separated human infants (ibid., p. 656).

McKinney & Bunney (1969) noted the behavioral similarities between these pigtail monkeys and the anaclitic depression of human infants described by Spitz (McKinney & Bunney, 1969, p. 242). Thus, they once again translated a psychiatric syndrome into behavioral terms and matched the behavior to the syndrome, evaluating, in modern terms, the face validity of the animal model.

### Psychopharmacology and animal models of depression in the 1950s and 1960s

As an anonymous referee has stressed, another important route to the creation of animal models in psychiatry was the rise of psychopharmacology, which related the established use of animal models in pharmacology to research conducted in psychiatry. In the following, we will consider how pharmacological research into the effects of reserpine led to the creation of one of the first animal models of depression. The reserpine model of depression was influential. In their 1969 article, McKinney and Bunney noted that the reserpine model was the most prevalent model of depression (p. 241). On the basis of a case study of the reserpine model of depression, we will see how research into psychopharmacology impacted the rise of animal models of mental disorders.

In an article entitled “Biogenic Amines and Emotion”, Schildkraut & Kety (1967), respectively a research scientist and director from the Laboratory of Clinical Science NIHM, observed that reserpine had been reported to cause severe depression in patients (p.24). These reports date back to the 1950s. For example, in 1957 Harris reported on a number of studies that showed that reserpine induced psychological side-effects such as anhedonia, depression, and suicide in patients (Harris, 1957). Harris cited authors who took such reserpine induced depressions to be indistinguishable from the usual depressions (Ibid.).

Schildkraut and Kety further noted that the effects of reserpine have been extensively studied in experimental animals (1967, p. 24). Through such studies it was established that reserpine leads to the depletion of tissue amine stores, such as the catecholamines and serotonin (ibid.). For example, in 1955, Pletscher, Shore & Brodie performed experiments on rabbits which showed that “reserpine effects the release of serotonin from the intestine, a major depot of serotonin in the body” (1955, p. 374).

The fact that reserpine induced depression and the biological knowledge obtained from experimental studies on animals pointed to the feasibility of an animal model of depression. In 1965, Brodie, citing the phenomenological similarities of the effects of reserpine and depression, took reserpine to allow the creation of an animal model of depression (Brodie, 1965, p. 129). In the 1960s, studies were published which concerned the reversal of the effects of reserpine. Costa et al., (1960) administered imipramine (an antidepressant) to rats before or after reserpine. Their experimental results “demonstrated that imipramine curtails selected pharmacological actions of reserpine including brain serotonin depletion” (1960, p. 463). Willner remarked on these studies that the reversal of the consequences of reserpine was “the earliest animal model of depression to be developed” (1984, p. 3).

Our study on the reserpine model of depression shows that animal experiments were common in psychopharmacology, where animals were used to study the effects of chemical substances. For example, the effects of reserpine were thoroughly studied in rabbits. Such experiments coupled with the observation that reserpine induced depression in humans led to new conceptualizations of the cause and nature of depression and gave rise to the idea that depression could be modelled via animals.

### Animal models as representations of clinical symptomatology

What was the role of manuals such as the *Diagnostic and Statistical Manual of Mental Disorders* (*DSM*) in evaluating animal models? This role becomes evident in a paper by Willner of 1991, entitled “Animal Models as Simulations of Depression”. This paper compared “the behavioral features of animal models of depression with clinical symptomatology” (Willner, 1991, p. 131).

The *DSM* in use in 1991 were the *DSM-III* of 1980 and the *DSM-III-R* of 1987. We have seen that the *DSM-I* and *DSM-II* were shaped by psychodynamic and psychoanalytic theoretical frameworks. The *DSM-III* was a revolution with respect to previous editions of the *DSM*. As First (2012b, p. 127) describes, one of the most significant innovations of the *DSM-III* was the introduction of operationalized diagnostic criteria for mental disorders. These criteria were meant to enhance the reliability and validity of diagnosis. In addition, the *DSM-III* adopted an “atheoretical approach” in which disorders are classified according to symptoms and not according to unproven etiological theories (First, 2012b, p. 132). In practice, this meant that the psychoanalytic and psychodynamic etiological theories which had dominated the *DSM-I* and *DSM-II* were excluded from the manual (133). First (2012b) notes the success of the *DSM-III*: it became “one of the most popular medical books ever published, with each edition selling well over a million copies” (First, 2012b, p. 136). When we study the use Willner makes of the *DSM-III*, we must keep in mind this popularity. This explains the crucial role Willner assigns to the DSM in evaluating animal models.

Willner (1991) compares the behavioral features of animal models of depression to the *DSM*. In order to demonstrate similarity between the behavior of animal models of depression and the clinical syndrome of depression, Willner cited the *DSM* criteria for diagnosing depression and indicated which features of depression could in principle be modeled in animals. Table 1 represents Willner’s construal of the depressive syndrome.

**Table 1:** Diagnosis of major depression (*DSM-III*, 1987, reconstructed with permission from Willner 1991, p. 133). Table reconstructed with permission from *Trends in Pharmacological Sciences* 12, Willner, P., Animal models as simulations of depression, 6 pages, 1991. With permission from Elsevier. 10.1016/0165-6147(91)90529-2

Requires the presence
Every (or nearly every) day
During the same two week period of
One core symptom and
Four subsidiary symptoms (or three with both core symptoms)
**Core symptoms**
Depressed mood
Loss of interest* or pleasure *
**Subsidiary symptoms**
Appetite disturbance* or weight change*
Insomnia* or hypersomnia*
Psychomotor retardation* or agitation*
Fatigue, or loss of energy*
Feelings of worthlessness, or excessive or inappropriate guilt
Decreased ability to think or concentrate*
Recurrent thoughts of death or suicide, or a specific suicide plan or attempt

* Could in principle be modeled in animals

After indicating which features of depression could be modeled by animals, Willner cited different animal models of depression and indicated which features of depression were present in the model. For example, the animal model of learned helplessness, originally established for dogs, exhibited decreased locomotor activity (D-LA), decreased motivation or persistence, and anhedonia (decreased response to rewards). Table 2 provides a partial reconstruction of Willner’s table of animal models of depression.

**Table 2:** Some animal models of depression (partially reconstructed from Willner 1991, p. 132). Table partially reconstructed with permission from *Trends in Pharmacological Sciences* 12, Willner, P., Animal models as simulations of depression, 6 pages, 1991. With permission from Elsevier. 10.1016/0165-6147(91)90529-2

Stress models	D-LA	I-LA	D-MP	IMP	D-SC	ANH
Learned Helplessness	+		+			+
Behavioral Despair and variants			+			
Failure to adapt to stress	+					
Chronic unpredictable stress	+					+
Separation models						
Primate separation models	+				+	
Distress calling in isolated chicks			+			

Legend: D-LA: decreased locomotor activity; I-LA: increased locomotor activity; D-MP: Decreased motivation/persistence. IMP: impulsivity. D-SC: decreased social contact; ANH: anhedonia (decreased response to rewards)

Willner’s methodology implies that we translate the syndrome of depression as described by the *DSM* into features that can be modeled in animals and subsequently check which features are modeled by different animal models.^5^ The method of Willner was clearly guided by the description of psychiatric syndromes given in the *DSM*, which is understandable given the huge impact of the *DSM-III* at the time. If we adopt this procedure, animal models are also validated in accordance with the *DSM*. As Willner put the point, remarking on the face validity of animal models, desirable features of an animal model of depression are that the model is phenomenologically similar to the syndrome and that the model captures core symptoms of depression (1991, pp. 131–132). In the next sections, we will see the problems that came to be associated with this approach

## Psychiatric genetics and criticisms of the *DSM* in the 1990s and 2000s

In the previous sections, I have argued that some scientists in the middle to late twentieth-century validated animal models of mental disorder by checking how well these animal models matched *DSM* classifications. In this section, I wish to argue that in the 1990s and 2000s, developments in psychiatric genetics went hand in hand with a critique of *DSM* classifications and a resultant skeptical view on attempts to validate animal models in terms of *DSM* categories. This provided the basis, as I will argue in the next section, for the articulation of the endophenotype approach to animal modeling.

During the 1990s there was a substantial increase in interest in research into the genetic basis of mental disorders, as can be seen from the funding landscape. Studying the National Institute of Mental Health (NIMH), Sadler remarks that “By the 1990s “Decade of the Brain,” the NIMH came to focus primarily upon funding basic neuroscience, genetic, and related research aimed at finding molecules and biomechanisms suitable for development of drug or other biomedical treatments.” (Sadler, 2013, p. 29). In 1998, the Genetics Workgroup of the NIMH recommended the “importance of accumulating the necessary infrastructure” as well as “close cooperation with other institutes, the training of more people with an interest in psychiatric genetics, and the establishment by NIMH of a Genetics advisory Group to monitor progress in this field (Barondes, 1999, p. 559). The director of the NIMH, Steven Hyman, noted that during his time as director (1996–2001) investment in human genetics grants increased from approximately $30 million a year to approximately $50 million a year, while genetic studies of animal studies were also extensively funded (2006, p. 109). What was the yield of this great investment?

Claussnitzer et al., (2020) provide a brief history of human disease genetics. They note that during the 1980s and 1990s efforts to identify genes for disease were largely focused on rare monogenic diseases. These efforts were driven by linkage analysis (Claussnitzer et al., 2020, p. 180). This enterprise was significantly helped by the completion of the draft human genome sequence (ibid.). However, Claussnitzer et al. also note that efforts to apply linkage analysis, which had been successful for Mendelian diseases, were largely unsuccessful for common traits with multifactorial aetiologies, such as depression (2020, p. 181). We can discern this historical development in publications of the 1990s. In 1990, the Yale geneticist Risch noted that several loci for several Mendelian disorders had already been identified and he claimed that the prospect of identifying loci for non-Mendelian diseases holds even greater promise (1990, p. 222). However, in 1996 Risch and Merikangas wrote that “the detection of genetic factors for complex diseases- such as schizophrenia, bipolar disorder, and diabetes- has been far more complicated” (1996, p. 1516). Risch and Merikangas note that few findings of genes for complex diseases have been replicated, which is partly explained by the modest nature of the gene effects for these disorders (Ibid.). According to Risch and Merikangas, linkage analysis, the method used for finding genes for rare and monogenic diseases, has “limited power to detect genes of modest effect” (Ibid.). Hence, they proposed a different method (association studies) that utilizes candidate genes, which has far greater power (ibid.). Risch and Merikangas concluded that “the future of the genetics of complex diseases is likely to require large-scale testing by association studies” (Ibid.).

As Nelson (2015) has described in detail, the 1990s were also the period when researchers had been able to create knockout mice. These new techniques, presented in 1992, were at first believed to enable researchers to make specific claims about the influence of individual genes on behavior (Nelson, 2015, p. 466). Experiments with knockout mice increased in the years after and lead to enthusiasm in the scientific and psychiatric community. Steven Hyman noted that in his tenure as director of the NIMH substantial investments went to areas such as transgenic and gene knockout mouse models (Hyman, 2006, p. 110). During this time we can, as an anonymous referee also stressed, witness the idea that novel genetic research could help carve nature at its joints and provide the basis for a new valid disease classification, supplanting the old and flawed *DSM*. Thus, to give an illustrative example, Krueger & Markon (2006) from the University of Minnesota argued that the *DSM* categories are not valid and that tying together findings from quantitative psychology, behaviour genetics, and personality psychology “provide the tools needed to develop an empirically based model of psychopathology” (2006, p. 114). With respect to behavior genetics, Krueger and Markon argued:

As our understanding of molecular neurobiology and genetics improves, it will also become possible to delineate the physical nature of the biological structures underlying psychopathology and its etiology. A greater understanding of the molecular-genetic substrates of psychopathology will help refine psychopathology models by providing details about the structures underlying the phenotypic organization of psychopathology. In this regard, molecular genetics not only helps explain why psychopathology occurs but also what psychopathology is- how it is best thought about and best organized conceptually. (Krueger & Markon, 2006, p. 115)

However, as Nelson describes, the results of knockout experiments soon became a subject of strong debate. Already in 1996, Genentech researcher Robert Gerlai noted the confounding effects of background genes, arguing that differences between mutant and control mice in knockout experiments were possibly due to genetic differences between the inbred strains used in the generation of the animals and not due to the mutation (Nelson, 2015, p. 475). In addition, there were, as Nelson describes, discussions about interpretations of knockout experiments, including on how to assess differing experimental reports. In a much cited study published in *Science* in 1999, researchers noted that there could be environmental conditions specific to different laboratories that lead to conflicting experimental reports on knockout experiments (Nelson, 2015, p. 478). These developments led to general scepsis toward the results of knockout experiments. In 2001, Gerlai noted that it was fundamentally problematic that knockout methods “took the individual gene as the primary unit of biological organisation, rather than systems of genes working in concert” (ibid., p. 479). Hence, within a decade knockout experiments were subject to extreme scrutiny and skepticism, and researchers could question whether genetic investigations could carve nature at its joints. As we will see below, other developments in psychiatric genetics in the 1990s and 2000s also lead to skepticism to the project of providing the biological foundations of psychopathology through genetic research.

What was the fate of the association studies that were proposed by Risch and Merikangas in 1996? Claussnitzer et al., (2020) note that early efforts at association studies with candidate genes were plagued by inadequate power and bias and confounding, resulting “in overblown claims and failed replication” (2020, p.181). These replication failures were mentioned, for example, by Hirschhorn et al., (2002). Hirschhorn was a researcher from the MIT Center for Genomic Research and the Department of Genetics Harvard, who together with co-authors reviewed association studies and noted that “most reported associations are not robust: of the 166 putative associations which have been studied three or more times, only 6 have been consistently replicated” (2002, p. 45). Ioannidis et al., (2001) similarly reported replication failings:

Here, we have evaluated by meta-analysis 370 studies addressing 36 genetic associations for various outcomes of disease. We show that significant between-study heterogeneity (diversity) is frequent, and that the results of the first study correlate only modestly with subsequent research on the same association. The first study often suggests a stronger genetic effect that is found by subsequent studies. Both bias and genuine population diversity might explain why early association studies tend to overestimate the disease protection or predisposition conferred by a genetic polymorphism. (Ioannidis et al., 2001, p. 306)

Hence, the early association studies in the 1990s and early 2000s did not provide robust findings. Early genome wide association studies also faced significant problems. In 2003, the HapMap Consortium developed “the first genome-wide maps of common sequence variation” (Claussnitzer et al., 2020, p. 187). In 2007, the first GWAS in psychiatric genetics was published (Arribas-Ayllon et al., 2019, p. 194). In 2005, researchers warned about the many false positives and the bias of population stratification in GWAS (Ibid.). By 2008, GWAS were criticized for the ‘missing heritability’ problem. This criticism cited “the disparity between heritability estimates and the combined genetic variance identified by GWAS results” (Arribas-Ayllon et al., 2019, p. 195). Hence, estimates of heritability “were being used to criticize the relatively poor yield from GWAS” (ibid.). Thus, for example, while height is taken to be highly heritable (80%), GWAS explained only 5% of this heritability (Ibid.).

Our story of psychiatric genetics in the 1990s and 2000s is a story of frustration. Although psychiatric genetics lead to important advances in psychiatry, finding genes for complex psychiatric diseases proved elusive. This led to skepticism about the idea that genetics could carve nature at its joints and provide the basis for a new classification of psychopathology. This frustration in psychiatric genetics provides a background for the emergence of the endophenotype project in psychiatry in the early 2000s, as I will discuss in the next section. This project proposed a new method of genetic analysis and promised great progress in linking genes to psychiatric diseases.

The frustrations with genetic psychiatry went hand in hand with criticisms of the *DSM*. In the following, I will describe some of these criticisms starting from the 1990s, focusing on criticisms that have been levelled against the *DSM* multiple times during recent decades, such as problems of heterogeneity and comorbidity. In 1990 Frances et al., presenting an overview of work in progress on DSM-IV, noted that many people reified the criteria in *DSM-III* despite the fact that most criteria were based on expert opinion (Frances et al., 1990, p. 1439). They further argued that the *DSM* did not provide an adequate definition of mental disorder and described the problem of comorbidity. They argued that a patient meeting criteria for more than one diagnosis may not have multiple independent conditions. Rather, co-occurring disorders may be part of a complex syndrome that has been “split apart in the *DSM* definition” (1990, p. 1443). There are also no sharp boundaries between conditions. This shed doubt on the categorical approach of the *DSM*, which assumes that members of a category are homogeneous and that categories are mutually exclusive. Finally, Frances et al. pointed to the heterogeneity of psychiatric diagnoses: “there are 93 different ways to meet the criteria for borderline personality disorder (76), and two patients may each meet the criteria for schizotypal personality without sharing even a single criterion for the diagnosis” (ibid., p. 1444).

Similar criticisms were voiced by Clark, Watson & Reynolds in 1995, from the Department of Psychology of Iowa. These authors argued that taxonomies can become unintended straightjackets (1995, p. 123). They noted that many writers reject the *DSM*’s attempt to be atheoretical with regard to the etiology of mental disorders, arguing that phenomenology is merely one way to classify disorders (p. 124). Like Frances et al., Clark, Watson & Reynolds argued that comorbidity is a major problem for the *DSM*. In some cases “comorbidity appears artifactual, reflecting the fact that the *DSM* allows two separate diagnoses to be assigned to what may be expressions of a single disorder” (p. 130). In addition, these authors highlighted, again like Frances et al., the heterogeneity of diagnoses: “the nine criteria for Borderline PD reflect a wide range of personality trait dimensions, from uncontrollable anger to identity disturbance. Two patients who meet both criteria for the diagnosis may share all nine criterion traits or they may share only a single one and exhibit rather different personality pathologies.” (p. 132).

The 2000s also witnessed a critique of the validity of the psychiatric diagnoses contained in the *DSM* and the *ICD*. This process was influenced by developments in psychiatric genetics, which we have reviewed above. As a result of these developments, researchers recognized that genetics could not provide a new classification of psychopathology. Hyman (2010), who as we have seen witnessed much research in psychiatric genetics as director of the NIMH, notes that psychiatric classifications in the *DSM* are useful heuristics, but that they are not valid and thus that we should not reify these mental disorders and treat them as natural kinds. In *DSM* and *ICD*, we rely on phenomenology as a basis for diagnosis. However, as Hyman notes, in disease classification, the gold standard is etiology (Hyman, 2010, p. 161). Since etiological information is still sparse, we are left with diagnoses that are not valid based on phenomenology. In addition, the prevalence of co-morbidity in psychiatric diagnoses may provide evidence for the invalidity of diagnostic practices, insofar as “co-morbidity might also reflect different patterns of symptoms that result from shared genetic risk factors” (Hyman, 2007, p. 727). Co-morbidity may reflect “the same underlying disease process”, or “a single pathophysiological process can cause symptoms that meet the criteria for multiple *DSM-IVTR* entries” (ibid.)

Within contemporary psychiatry the characterization of mental disorders in terms of phenomenological signs and symptoms, which is characteristic of classification manuals such as the *DSM* is often problematized. The objection to understanding mental disorders merely in terms of phenomenological signs and symptoms is that such an understanding of mental disorders does not reflect the causes of mental disorders and thus does not provide us with a proper understanding of mental disorders. This line of reasoning takes diagnoses to be valid if they reflect causal structures: a diagnosis “is valid if it rests on a biological process that can be identified by experiment and observation using the methods of the biological and cognitive sciences” (Murphy, 2017). This definition of a valid diagnosis resembles the definition of Hyman (2007, p. 730), who argues that a valid diagnosis is “a diagnosis that picks out a real entity based on aetiology or pathophysiology”.

The stress on the importance of identifying the etiology of mental disorders in order to increase the validity of diagnoses can be found in the National Institute of Mental Health’s (NIMH) proposal to introduce the Research Domain Criteria (RDoC) in grant proposals (Insel et al., 2010), which arose after early genetic research had not been able to identify the biological basis or causes of psychopathology. As Insel et al. explain, the NIMH introduced the RDoC in grant proposals to create a framework for pathophysiology that will influence future classification schemes (Insel et al., 2010, 748). Insel et al. note that current classification schemes have increased the reliability of psychiatric diagnoses, but not their validity, since the diagnostic categories used within the *DSM* and *ICD* do not capture mechanisms of dysfunction (ibid.). Valid diagnostic categories should reflect such underlying mechanisms.

Commenting on RDoC, First, a clinical psychiatrist who was consultant for the DSM V, notes that the *DSM* and *ICD* have been unsuccessful in facilitating the causal mechanisms behind mental illness (First, 2012a, p.13). Moreover, he notes that many psychiatrists now think that the *DSM* is holding research into the mechanisms of mental illness back (ibid., p.14. See also Kaffman & Krystal, 2012). RDoC aims to develop a new diagnostic framework based on neuroscience and genetics (First, 2012a, p. 15). The overarching aim of RDoC is to develop new methods for classifying mental disorders based on observable behavior and neurobiological data (First, 2012a, p. 15). The RDoC project specifies “basic dimensions of psychological functioning and their corresponding brain circuits that have been the focus of neuroscience research over the past decades” (ibid.). RDoC is, as First notes, primarily a project aimed at furthering research. The goal is to provide a framework that allows researchers to relate domains of behavioral functioning to underlying neurobiological components (ibid.). According to First “The RDoC approach represents a true paradigm shift in the classification of mental disorders, moving away from defining disorders based on descriptive phenomenology and instead focusing on disruptions in neural circuitry as the fundamental classificatory principle” (First, 2012a, p. 16).

## The endophenotype approach to animal modeling

In the early twenty-first century the representational target of animal models shifted. Rather than modeling psychiatric disorders, researchers now often believe that animal models model selected components of mental disorders. Such components are called endophenotypes and researchers who model endophenotypes adopt a so-called endophenotype approach (Gottesman & Gould, 2003). The basic idea of the endophenotype approach is summarized by Fernando & Robbins, from the Behavioural and Clinical Neuroscience Institute and Department of Experimental Psychology Cambridge, who claim that it is unwise to model entire syndromes and that we must “focus instead on well-understood symptoms or symptom clusters” (2011, p. 40). In the present section, I explain the endophenotype approach and its appeal to early twenty-first century researchers.

In her *Animal Behavior* (2018), Nelson mentions that the main challenges confronting animal researchers of mental disorders were the complexity of human behavior and disorders, the complexity of animal behavior, and the complexity of the genetic and environmental factors that influence both animal and human behavior (2018, pp. 21–22). In order to cope with this complexity, Nelson argues that researchers adopted the experimental practice of “breaking down behaviors into smaller units for analysis and creating controlled laboratory environments in which to study them” (2018, p. 23). By adopting this methodology, animal behavior “could be manipulated and segmented in ways that would be impossible in humans” (2018, p. 28). Decomposing mental disorders into component parts was, Nelson argues, part of the endophenotype project, which was both an epistemological and ontological project. In her very brief description of the endophenotype project, Nelson states that the endophenotype project was ontological because it proposed to “reorganize existing psychiatric disease categories” and establish a biological basis for diagnosis (2018, p. 29). Nelson argues that this belief was “far from a central tenant of behavior genetics research” (ibid.). Rather, researchers adopted the project of decomposing mental disorders into smaller subunits because it was a viable methodological practice. The reductionist approach or the method of partial modeling was simply a sure way to generate knowledge of complex phenomena and it was “an experimental strategy rather than an ontological commitment” (ibid.).

Nelson’s analysis of partial modeling is instructive. It is no doubt true that part of the appeal of adopting the reductionist approach of decomposing mental disorders into smaller subunits is that it is a useful methodological practice. This programmatic view on animal modeling predates the endophenotype project. Already in 1977, Hanin and Usdin stated that “one cannot reproduce the exact syndrome in an animal that one is trying to mimic from man” (p. xiii). In 1988 McKinney similarly wrote that “we now recognize there is no such thing as a comprehensive animal model for any psychiatric syndrome” (McKinney, 1988, p. 2). Rather, we study selected aspects or components of human psychopathology (ibid.). This is taken to be a better characterization of what animal models of mental disorder actually represent. In 2015, Willner and Belzung also write: “even if researchers often have the explicit intention to develop models of a pathology, the reality is that these models are typically limited in scope: they simulate specific aspects […] rather than the entirety of the disorder” (2015, p. 3474).

However, the popularity of the endophenotype approach, as Nelson also recognizes, cannot be fully explained in terms of this pragmatic stance. Rather, we must take into account the historical circumstances sketched in the previous sections. There we described that psychiatrists increasingly criticized the *DSM* in the 1990s and 2000s and we described the problems faced by psychiatric genetics in identifying genes for complex mental disorders. It is these factors that explain the rise of the endophenotype project at the start of the twenty-first century and the abandonment of the *DSM* as a validator of animal models. Moreover, animal researchers endorsed the endophenotype approach because it allows them to create more *valid* animal models. This point, which Nelson also does not consider, is crucial for understanding the popularity of the endophenotype approach. For example, according to Fernando & Robbins, modeling entire disorders is “confounded by the heterogeneous nature of symptoms of psychiatric disorders, their high level of comorbidity, and a sometimes misguided adherence” to the DSM” (2011, p. 40). Through modeling endophenotypes or components of disorders we will “enhance model specificity and validity” (2011, p. 41). In the following, I explain the endophenotype approach and describe why researchers think modeling endophenotypes allows us to better validate animal models.

An endophenotype, as explained by Gottesman & Gould (2003), geneticists from the Department of Psychiatry University of Minnesota, is defined as a measurable component “unseen by the unaided eye along the pathway between disease and distal genotype” (p. 636). The term was introduced in a 1966 paper by John and Lewis, who used endophenotypes to explain the geographical distribution of grasshoppers (Gottesman & Gould, 2003, p. 637). Endophenotypes were described as “microscopic and internal traits”, and were contrasted with exophenotypes, which were “obvious and external traits” (e.g., behavior or physical appearance) (Glahn et al., 2014).

Characteristic of the endophenotype approach within psychiatry is that it is analytic: it involves decomposing complex behaviors and syndromes into their component parts or endophenotypes (Lenox et al., 2002, p. 391). Psychiatric syndromes are conceptualized as complexes of core symptoms which can, if they satisfy a certain set of conditions, be endophenotypes (Panksepp, 2006, p. 775). Endophenotypes themselves are “abnormal neurophysiological, biochemical, endocrinological, neuroanatomical, cognitive and neuropsychological findings” that often accompany psychiatric illness (Lenox et al., 2002, p. 392). For example, the complex syndrome schizophrenia, which is characterized by positive symptoms (such as hallucinations) and negative symptoms (such as social withdrawal) can be construed as having endophenotypes such as: deficits in sensory motor gating, impaired working memory, eye tracking dysfunction, and so forth (Gottesman & Gould, 2003).

The rationale for decomposing psychiatric syndromes into endophenotypes is that it enables more straightforward and successful genetic analyses (ibid.). Gottesman & Gould (2003) explain the rationale for the endophenotype approach as follows:

This rationale held that if the phenotypes associated with a disorder are very specialized and represent relatively straightforward and putatively more elementary phenomena (as opposed to behavioral macros), the number of genes required to produce variations in these traits may be fewer than those involved in producing a psychiatric diagnostic entity. (Gottesman & Gould, 2003, p. 637)

Hence, while it is difficult to identify the genetic underpinnings of psychiatric diagnostic entities, it is more straightforward to identify the genetic underpinnings of more elementary components of diagnostic entities. For this reason, researchers think that the endophenotype approach facilitates genetic analysis and makes mechanistic insight into mental disorders more tractable than analyses that focus on the mechanisms responsible for whole psychiatric diagnostic entities (Arguello & Gogos, 2006). This aspect of endophenotypes must have been attractive to researchers familiar with the frustrations of psychiatric genetics in the 1990s and 2000s. In addition, this aspect of endophenotypes distinguishes the endophenotype approach from the partial modelling described by Nelson. As an anonymous referee has stressed, the partial modelling approach described by Nelson involves a pragmatic and modest stance on what animal models could do, acknowledging that modeling complex disorders is not feasible, whereas the endophenotype approach is meant to identify new phenomena between genes and complex disorders that facilitate genetic analysis.

Apart from facilitating research into gene linkage studies, researchers argue that by adopting the endophenotype approach we can better *validate* animal models (see Gould & Gottesman, 2006; Gottesman & Gould, 2003; Lenox et al., 2002; Fernando & Robbins, 2011. Anderzhanova et al., 2017). We can understand this view better if we understand why contemporary researchers think the old idea that animal models represent *DSM* categories leads to problems with validating animal models of mental disorders. Several problems associated with validating animal models on the basis of how well they approximate *DSM* disorders are illustrated by two skeptical objections that Nestler & Hyman (2010) articulate against animal models. First, Nestler and Hyman argue that it is difficult to determine how symptoms in an animal add up to a human disorder given the inexact state of psychiatric diagnosis in humans (Nestler & Hyman, 2010, pp. 1161–1162. See also Kaffman & Krystal, 2012). Nestler and Hyman, stressing the heterogeneity of psychiatric diagnoses that was also stressed by critics of the *DSM* in the 1990s and 2000s, note that two individuals can both be diagnosed with depression according to the *DSM* without sharing any symptoms. The fact that different individuals can be diagnosed with depression without any overlap in their symptoms means that different animal models of depression may have little in common. Similar problems arise for other mental disorders. This state of affairs creates difficulties for *validating* animal models. Given the imprecise state of psychiatric diagnosis in humans, it is not clear on the basis of which signs and symptoms we can argue that an animal model has achieved an acceptable level of face validity (Nestler & Hyman, 2010, p. 1162).

Similar problems arise with respect to judging the construct validity of animal models of mental disorders. Nestler and Hyman note that a simple way of achieving construct validity would be to insert a disease-causing genetic mutation into, say, a mouse or inserting a penetrant genetic variant that increases vulnerability to a human disease. However, this is not currently possible “as such disease-causing genes have not been established with certainty and most disorders exhibit highly complex genetic architecture” (Nestler & Hyman, 2010, p. 1162. See also Anderzhanova et al., 2017, p. 48; Kaffman & Krystal, 2012). Similarly, we cannot achieve construct validity through exposure to known environmental risk factors, since “virtually all environmental contributions to mental illness, such as stress or childhood adversity, are associated with multiple disorders and most often normal outcomes” (Nestler & Hyman, 2010, p. 1163). Since we cannot establish unique links between specific environmental risk factors and specific mental disorders, we cannot decisively argue for the construct validity of animal models of some particular disorder.

Note that both objections center on the fact that it is difficult to establish unique links between animal models of mental disorders and classifications of diseases given in manuals such as the *DSM*. This creates a problem in establishing the face and construct validity of animal models. However, rather than taking these problems as objections to the process of building animal models of mental disorders, many researchers conclude that it is problematic to validate animal models by judging how well they approximate categories of diseases listed in manuals such as the *DSM*. This is the position of Nestler and Hyman, who argue that researchers “must rely on judgment rather than slavish devotion to meeting all DSM-IVTR criteria for the disorder being modeled” (Nestler & Hyman, 2010, p. 1161). A similar position is taken by Lemoine, who argues that animal models of depression are problematic because of the fuzzy definition of depression and because of the difficulty of “linking together supposedly involved biological mechanisms into a consistent picture of the underlying process of the disease (Lemoine, 2015, p. 157).

We can conclude, as we have already seen, that there is widespread consensus that the disease classifications contained in manuals such as the *DSM* and *ICD* are not valid. The lack of validity of current diagnostic categories provided researchers with a reason to be skeptical of attempts to validate animal models of mental disorder by looking at whether the animal models match syndromes described in the *DSM* and *ICD*. For example, Kaffman & Krystal (2012) argue that reliance on the *DSM* hinders the progress of the development of animal models and note that the *DSM* provides an inadequate framework for developing animal models with predictive and construct validity. For this reason, they argue that we should abandon the *DSM* as a standard for validating animal models of mental disorders.

How does the endophenotype approach allow for the creation of more valid animal models of mental disorder according to proponents of this approach? Gould & Gottesman (2006) note that instead of validating animal models in terms of heterogeneous disease entities, endophenotypes represent “more defined and quantifiable features” (p. 115). While we cannot model a complex heterogeneous disease entity, endophenotypes are “proving more amenable to the task” (p. 116). Animal models of endophenotypes are more straightforward and congruent with the human condition at the level of biology and genetics and are thus better validated (Ibid.). Hence, we can more easily find animal analogs of human conditions at the level of endophenotypes. The idea is further that since the endophenotype approach makes genetic analysis of the genes underlying endophenotypes more tractable, we are better capable of specifying the biological causes of these endophenotypes and are thus capable of constructing animal models with high construct validity. Thus, Gould and Gottesman remark optimistically with respect to schizophrenia that the “cause of putative neuroanatomical and neurodevelopmental endophenotypes in schizophrenia […] may soon be explained as the functional consequences of susceptibility genes are elucidated” (Ibid.). In this way, endophenotypes better allow us to create animal models with construct validity. Gould and Gottesman note that since we model endophenotypes, and not complex diseases, the face validity of animal models of endophenotypes may be low if we compare the animal model to a complex disease classification in the *DSM* (p. 117). However, they think that if we compare an animal model to endophenotypes, which they define as more well-defined and measurable features than heterogeneous disease entities, it is also possible to construct animal models with high face validity. A similar approach is advocated by Fernando & Robbins (2011). Commenting on Obsessive-Compulsive disorder, they note that it is possible to define endophenotypes of this disorder, such as impairment in stop signal inhibition and extradimensional set-shifting (a task involving perceptual discriminations) (p.47). Fernando and Robbins argue that these endophenotypes “have analogs in experimental animals” (ibid.), and by relating these endophenotypes to their (in this case) neurological causes we can achieve animal models with high construct validity.

For the above reasons, researchers argue that we can better validate animal models if we model endophenotypes then if we model heterogeneous disease entities (Gould & Gottesman, 2006; Gottesman & Gould, 2003; Lenox et al., 2002; Fernando & Robbins, 2011). As Lenox, Gould and Manji put the point: “Defining endophenotypes will result in the potential to create animal models with both face and construct validity linked to the disease in the human and will permit intensive scientific investigation into the underlying biology of the endophenotype under investigation” (2002, p. 401). Insofar as endophenotypes provide insight into the etiology of mental disorders, it is argued that they further allow us to provide valid disease classifications. Gould & Gottesman (2006) argue that endophenotypes will aid with diagnosis and classification (p. 115). The idea that endophenotypes yield valid disease categories again demonstrates a difference between the partial modeling approach described by Nelson and the endophenotype approach. The latter allows us to provide, as an anonymous referee stressed, biologically grounded and valid disease categories that allow us to validate animal models, whereas partial modelers were less committed to an agenda of providing valid disease categories.

To conclude: in the first decade of the twenty-first century, endophenotypes became regarded as validators of animal models of mental disorder. The focus on endophenotypes allows us, it is argued, to better validate animal models, makes use of more valid categories, and makes tractable the discovery of the mechanisms responsible for mental disorders. As such, the endophenotype approach is taken to be one of the prime methods for achieving face and construct validity of animal models. The rejection of diagnostic categories as listed in the *DSM* as validators of animal models, the focus on endophenotypes, and the importance of the etiology and mechanisms responsible for endophenotypes and mental disorders is characteristic of present day psychiatry and the RDoC approach. This is aptly summarized by Kaffman & Krystal (2012), from the Department of Psychiatry of Yale, who describe their own approach to animal modeling as follows:

First, it proposes to replace the *diagnostic and statistical manual of mental disorders* (DSM) diagnostic system with measurable endophenotypes as the basis for modeling human psychopathology in animals. We argue that a major difficulty in establishing valid animal models lies in their reliance on the DSM/*International Classification of Diseases* conceptual framework, and suggest that the Research Domain Criteria project, recently proposed by the NIMH, provides a more suitable system to model human psychopathology in animals. (Kaffman & Krystal, 2012, p. 3. As quoted in Miller & Rockstroh, 2013, p. 202. See for a similar point Anderzhanova et al., 2017)

## Conclusion

The issue of validity is crucial in the history and philosophy of animal modeling. This paper highlights different attempts at constructing valid animal models of mental disorders, focusing on animal models of depression. After explaining the criteria on the basis of which animal models of mental disorders are validated, I have described a shift in the representational target of animal models of mental disorders: the shift from the view that animals model psychiatric syndromes as classified in manuals such as the *DSM* to the view that animals model component parts of such syndromes. This shift leads to different ways of validating animal models of mental disorders. The view that animal models model psychiatric syndromes defined in manuals such as the *DSM* was adopted during the 1960s, when the cooperation between psychoanalysis, comparative psychology and ethology as well as developments within psychopharmacology lead to the creation of animal models of mental disorders, up to the 1990s, at the heights of the popularity of the *DSM-III*. This view was often abandoned after challenges facing psychiatric genetics and increasing criticism of the *DSM* in the 1990s and 2000s. Accordingly, many researchers adopted the so-called endophenotype approach to animal modeling, which holds that animal models model parts of mental disorders. Several researchers believe that this approach allows them to better validate animal models, even if the fruitfulness of the endophenotype approach remains to be proven.

## Data Availability

Not applicable.

## Footnotes

1 Related work on animal modeling in medicine, which I found through Ankeny et al., (2014), is contained in Bynum (1990), Löwy (1992), Friese & Clarke (2011) and Germain (2014). On problems associated with animal modeling and experimentation, see Lafollette & Shanks (1996) and Degeling & Johnson (2013). The standardization of animal models is a core question in animal modeling (see Ankeny et al., 2014, pp. 497–499. See Rader (2004) on standardization and mice). However, it falls outside the scope of the present paper.

2 For a systematic analysis of the different meanings of construct validity, see Slaney 2017.

5 As an anonymous referee pointed out, the fact that Willner compared different animal models gives rise to some difficulties. As the referee argued, different tests done on different species can reflect different processes. For this reason, it is not clear, for example, that a dog’s learned helplessness entails the same decreased motivation (“D-MP”) as a chick’s distress calls. I use Willner’s table here merely to highlight the role of manuals such as the *DSM* in validating animal models.
